# Hydrogen Sulfide Donor NaHS Improves Metabolism and Reduces Muscle Atrophy in Type 2 Diabetes: Implication for Understanding Sarcopenic Pathophysiology

**DOI:** 10.1155/2018/6825452

**Published:** 2018-10-30

**Authors:** Milad S. Bitar, Joelle Nader, Waleed Al-Ali, Ashraf Al Madhoun, Hossein Arefanian, Fahd Al-Mulla

**Affiliations:** ^1^Department of Pharmacology & Toxicology, Kuwait University, Faculty of Medicine, Kuwait; ^2^Immunology Unit, Dasman Diabetes Institute, Kuwait; ^3^Department of Mathematics & Natural Sciences, American University of Kuwait, Kuwait; ^4^Department of Pathology, Kuwait University, Faculty of Medicine, Kuwait; ^5^Functional Genomics Unit, Dasman Diabetes Institute, Kuwait

## Abstract

Sarcopenia, a loss of muscle mass and functionality, constitutes a major contributor to disability in diabetes. Hydrogen sulfide (H_2_S) dynamics and muscle mass regulatory signaling were studied in GK rats, a model for type 2 diabetes (T2D). GK rats exhibited a number of features that are consistent with sarcopenia and T2D including loss of muscle mass and strength, in addition to glucose intolerance, insulin resistance, and impaired *β*-cell responsiveness to glucose. Mechanistically, activation levels of Akt, a key modulator of protein balance, were decreased in T2D. Consequently, we confirmed reduced activity of mTOR signaling components and higher expression of atrophy-related markers typified by FoxO1/atrogin-1/MuRF1 and myostatin-Smad2/3 signaling during the course of diabetes. We observed in GK rat reduced antioxidant capacity (↓GSH/GSSG) and increased expression and activity of NADPH oxidase in connection with augmented rate of oxidation of lipids, proteins, and DNA. H_2_S bioavailability and the expression of key enzymes involved in its synthesis were suppressed as a function of diabetes. Interestingly, GK rats receiving NaHS displayed increased muscle Akt/mTOR signaling and decreased expression of myostatin and the FoxO1/MuRF1/atrogin-dependent pathway. Moreover, diabetes-induced heightened state of oxidative stress was also ameliorated in response to NaHS therapy. Overall, the current data support the notion that a relationship exists between sarcopenia, heightened state of oxidative stress, and H_2_S deficiency at least in the context of diabetes. Moreover, treatment with a potent H_2_S donor at an early stage of diabetes is likely to mitigate the development of sarcopenia/frailty and predictably reduces its devastating sequelae of amputation.

## 1. Introduction

Sarcopenia, a loss of muscle mass and function, is one of the most dramatic phenotypic changes during the course of advanced aging [1, 2] and diabetes. It contributes to poorer glycemic control, decreased muscle glucose uptake, insulin resistance, disability, and frailty [3]. Moreover, recent data indicated that sarcopenia is an important prognostic factor for critical limb ischemia (CLI) [4]. The latter phenomenon is a manifestation of peripheral arterial disease, affecting nearly 2 million people in United States, and it is usually described in terms of chronic ischemic rest pain or ischemic skin lesions with either ulcers or gangrene [5, 6]. Indeed, patients with CLI and diabetes have poor prognosis in connection with severe atherosclerotic lesions and high risk of amputation [7–11].

Sarcopenia is relatively easy to diagnose by preoperative computed tomography imaging, and a relationship between this pathology and CLI has recently been established focusing on the notion that sarcopenia by altering key molecules such as adiponectin and carnitine exacerbates whole-body arteriosclerosis, henceforth contributing to the poor prognosis of CLI patients [12]. The causes of sarcopenia are yet to be illustrated but may include hormonal imbalance, denervation, mitochondrial dysfunction, inflammation, oxidative stress, and decreased gasotransmitter bioavailability. Most of the aforementioned pathogenetic processes are altered as a function of diabetes and advanced aging. In view of this information, it is reasonable to suggest that improving the prognosis of CLI is achievable by the early recognition and target therapeutic of sarcopenia as a whole-body disease.

Skeletal muscle is now considered to be an endocrine organ, synthesizing and secreting a variety of bioactive molecules including inflammatory cytokines, growth factors, adipokines, carnitine [13], and more recently hydrogen sulfide (H_2_S) [14]. H_2_S has emerged as a critical inter- and intracellular signaling molecule similar to NO and CO with a profound impact on metabolic, inflammatory, and neurovascular processes [15]. This novel gasotransmitter is endogenously produced from L-cysteine or L-methionine by three main enzymatic contributors: cystathionine *γ*-lyase (CSE), cystathionine *β*-synthase (CBS), and 3-mercaptopyruvate sulfurtransferase (3-MST). CSE and CBS have been shown to localize in cytoplasm whereas 3-MST is found mainly in the mitochondria [16]. All of the three enzymes are expressed in human and rat skeletal muscles [14].

A panoply of evidence indicates that H_2_S exhibits anti-inflammatory, antioxidant, and antiatherosclerotic properties [15]. In this context, H_2_S appears to provide protection against low oxygen and nutrient supply as well as ischemic injury in multiple organs including skeletal muscles [14, 17, 18]. This beneficial effect stems from H_2_S's ability to attenuate the excessive generation of reactive oxygen species and thereby preventing neuronal degeneration and vascular and skeletal muscle injury [19]. Similarly, recent studies have confirmed that H_2_S plays an antiatherosclerotic role, and its deficiency leads to early development and progression of atherosclerosis [20]. Interestingly, high fructose diet-induced type 2 diabetes in rats was found to associate with a decline in serum H_2_S level and supplementation with H_2_S improved insulin sensitivity, reduced triglyceride levels, and enhanced oxidative stress tolerance [21]. In view of the above information suggesting a favorable effect of H_2_S on a number of pathogenetic parameters known to be involved in the development of sarcopenia, arteriosclerosis, and CLI, we initiated the current study to, firstly, investigate in the context of diabetes the critical determinants of muscle mass homeostasis as typified by Akt-mTOR-, myostatin-Smad2/3-, and FoxO1-atrogin-dependent pathways, secondly, examine the impact of the diabetic state on skeletal muscle endogenous H_2_S dynamics, and thirdly, highlight the therapeutic effectiveness of the H_2_S donor in ameliorating the development of muscle wasting and counteracting its progression during the course of diabetes.

## 2. Materials and Methods

### 2.1. Animals

Goto Kakizaki (GK; age 12–15 months) female rats weighing 275–325 g, a model for nonobese type 2 diabetes [22, 23], and their Wistar control counterparts with a similar body weight were used for this experiment. In addition, we also included in our experimental groups 4-month and 26-month-old rats, typifying, respectively, the young and advanced aging. The animals were housed under diurnal lighting conditions and fed on a laboratory diet with water. Animal experiments were conducted according to the guidelines for Principles of Laboratory Animal Care and the Guide for the Care and Use of Laboratory Animals (NIH publication no. 85-23, revised 1996). The current investigation was approved by the local institutional review board of Kuwait University, Faculty of Medicine.

### 2.2. Treatment

A group of diabetic rats was injected daily by an intraperitoneal route with the H_2_S donor NaHS at a dose of 5.6 mg/kg body weight for a duration of 8 weeks; the corresponding vehicle-treated normoglycemic control and diabetic animals received saline alone.

### 2.3. Glucose (OGTT)/Insulin Tolerance Tests

Rats derived from various experimental groups were orally administered with 50% glucose at a dose of 1.5 g/kg or IP injection with insulin at a dose of 0.8 IU/kg. Tail vein blood samples were collected at 0, 30, 60, 90, and 120 min for OGTT or at 0, 5, 10, 20, and 30 min for ITT and then centrifuged (6000 x g, 4°C) for 15 min to get the serum samples for glucose and insulin assays. Homeostasis assessment of *β*-cell function (HOMA-*β*) was calculated using the following formula: [insulin (*μ*U/ml) × 20]/[glucose (mmol/l) − 3.5]. Values represent fasting insulin and glucose levels [24].

### 2.4. Blood and Skeletal Muscle Collection and Functionality Measure

Animals were allowed 3 days of recovery following the OGGT/ITT, fasted overnight, and then anesthetized using an IP injection of ketamine/xylazine prior to sacrifice by decapitation. Blood was obtained and centrifuged (6000 x g, 4°C, and 15 min), and serum samples were collected for biochemical assays. Gastrocnemius (GN) and soleus (SOL) muscles were rapidly dissected on ice, weighed, and flash frozen. All samples were stored at −80°C until further analysis. A grip strength test was conducted according to previously published procedure [25].

### 2.5. Tissue Preparation

Frozen muscles were powdered under liquid nitrogen, submerged in ice-cold RIPA buffer containing a cocktail of protease and phosphatase inhibitors (Sigma, USA), homogenized at 0–4°C with a tissue grinder (Tekmar, Germany) for 15 s, and centrifuged at 16,000 x g for 15 min, and then the supernatants were used as a source for total proteins. The nuclear protein extraction (NE-PER) kit (Thermo Fisher Scientific, USA) was used according to the manufacturer's instruction to separate the cytosolic and nuclear fractions. Mitochondria were isolated by differential centrifugation according to the manufacturer's description (Thermo Fisher Scientific, USA). Briefly, reagent C was added and centrifuged at 700 x g for 10 min at 4°C. The resulting pellets containing unbroken cells and nucleus were discarded, and the supernatant was transferred to new Eppendorf tubes and further centrifuged at 3000 x g to obtain mitochondrial fraction.

### 2.6. Assessment of Redox Status

#### 2.6.1. Dihydroethidium (DHE) Staining

Frozen muscle sections (10 *μ*m thickness) were incubated with DHE (5 *μ*mol/l) for a duration of 30 min. After staining, sections were examined with an LSM 5101 confocal laser scanning microscope (Carl Zeiss) and Axiovert 100M was used to analyze and capture representative images.

#### 2.6.2. NADPH Oxidase Activity

NADPH oxidase dependent-superoxide anion generation in muscle microsomal fractions was measured using either the lucigenin (5 *μ*mol/l) chemiluminescence-based [26] or the Amplex Red (50 *μ*mol/l, Molecular Probes, USA) horseradish peroxidase (0.5 U/ml, Roche, USA) fluorescence-based assays [27]. The specificity of superoxide measured was confirmed by adding peg-superoxide dismutase (SOD; 10 U/ml).

#### 2.6.3. Macromolecule Oxidative Markers

Thiobarbituric acid-reactive substances (TBARSs), reflecting lipid peroxidation, were measured according to previously published procedure [28]. Levels of protein-bound carbonyls representing total protein oxidation and 8-hydroxy-2-deoxyguanosine (8-OHdG) in plasma or muscles were determined using commercially available kits provided by BioVision and Enzo Life Sciences, USA, respectively.

#### 2.6.4. Glutathione Antioxidant Defense System

Total and oxidized glutathione (GSH and GSSG) contents in tissue homogenates were quantified as described previously [29]. Protein was precipitated with sulfosalicylic acid, and the GSH level was measured as the yellow derivative of 5-thio-2-nitrobenzoic acid in the presence of NADPH and 5,5-dithiobis(2-nitrobenzoic acid). To measure GSSG concentration, 2-vinyl pyridine was added to chelate GSH.

Glutathione reductase (GR) activity was determined by the spectrophotometric disappearance of NADPH at 340 nm using a cocktail solution containing 10 mM NADPH, 30 mM GSSG, 200 mM MOPS, 620 mM sucrose, 1 mM EDTA, and 30 mM thiourea [30]. Values were expressed as nmol/mg protein/min.

#### 2.6.5. Cystathionine *γ*-Lyase (CSE)

Activity of CSE was assessed according to published procedures [31, 32]. The assay involves adding tissue homogenates to an assay buffer containing 400 mM Tris, 40 mM ATP, 40 mM L-glutamic acid, 2 mM EDTA, 20 mM sodium borate, 2 mM serine, and 40 mM MgCl_2_. Protein was precipitated with sulfosalicylic acid and the supernatant was reacted with 2,3-naphthalenedicarboxyaldehyde (NDA), and the formation of NDA-*γ*-GC was measured (472 nm excitation/528 nm emission) using a fluorescent plate reader (Promega, USA).

#### 2.6.6. Mitochondrial ROS Production and Complex Activity

H_2_O_2_ production in isolated mitochondria was measured using the Amplex Red-horseradish peroxidase method (Molecular Probes, USA). A similar result was also obtained using a previously published fluorescence-based assay [33].

The enzymatic activity of mitochondrial complex 1 (NADH: ubiquinone oxidoreductase) and complex III (decylubiquinol cytochrome c oxidoreductase) were measured in isolated mitochondria using previously published procedures [34, 35].

#### 2.6.7. Plasma Myostatin

Plasma myostatin level was determined using commercially available rat ELISA-based assay (MyBioSource, USA).

### 2.7. Assessment of H_2_S Levels and H_2_S-Producing Capacity in Gastrocnemius Muscles and in Serum

H_2_S levels in serum and muscle tissue were measured according to previously published procedure with some modifications [36]. Muscle was homogenized in ice-cold Tris-HCl buffer (100 mmol/l, pH 8.5) with protease inhibitors, followed by centrifugation at 12,000 x g for 20 min at 4°C. 100 *μ*l of muscle homogenate or serum was added to premixed 100 *μ*l 1% zinc acetate/3 N NaOH *μ*l, placed on ice for 5 min, and then centrifuged at 1500 x g for 10 min. The supernatant was discarded, and the pellet containing zinc sulfide was reconstituted with 100 *μ*l of H_2_O and then reacted with a premixed dye containing 20 mmol/l of N,N-dimethyl-p-phenylenediamine sulfate dissolved in 7.2 N HCl and 30 mmol/l FeCl_3_ in 1.2 N HCl. The reaction mixture was incubated for 10 min to allow for color development, and then it was read on a spectrophotometer at 670 nm.

Tissue H_2_S producing capacity was measured as described previously with slight modification [37]. Briefly, phosphate buffer (100 mmol/l, pH 7.4)-based homogenates 500 *μ*l (*w*/*v*, 1 : 10) were incubated with L-cysteine (10 mmol/l; 25 *μ*l), pyridoxal 5-phosphate (1 mmol/l; 25 *μ*l), and PBS (25 *μ*l) in a tightly sealed Eppendorf tube for a duration of 90 min at 37°C. Subsequent steps were conducted as described above for the measurement of basal level of H_2_S. H_2_S concentrations were assessed using a curve generated with NaHS (0–200 *μ*mol/l) standards. Total protein levels were determined using BCA reagent kit (Pierce, USA).

### 2.8. Quantitative Real-Time PCR

Total RNA from gastrocnemius muscles was extracted using the TRIzol reagent (Invitrogen, USA), and RNA integrity was verified by agarose gel electrophoresis. Approximately 1 *μ*g of RNA was reverse-transcribed (Superscript II Reverse Transcriptase Kit, Invitrogen) and amplified using the TaqMan Assay on Demand (Applied Biosystems, USA) in a 25 *μ*l reaction volume containing two unlabeled primers, a 6-carboxyfluorescein-labeled TaqMan MGB probe and the master mix. The amplified sequences were assessed using the ABI 7500 Prism Sequence Detection system machine. The results were expressed as mRNA levels normalized to 18S or GAPDH in each sample. qPCR was conducted for miR-486 with gene expression assays using specific TaqMan miR assays (Applied Biosystems, USA). U6 was used as a reference gene.

### 2.9. Antibodies

Primary antibodies used in the current study included the following: rabbit anti-PTEN, 9552; rabbit ant-phospho S6 (Ser235/236), 4858; S6, 2217, rabbit anti-phospho-4E-BP1 (Thr37/46), 2855; rabbit anti-phospho-Akt (Ser473), 4060; rabbit anti-phospho-Akt (Thr308), 4056; rabbit anti-Akt, 9272; rabbit anti-FoxO1, 2880; rabbit anti-phospho-FoxO1 (Ser256), 8419; rabbit anti-Smad2, 5339; rabbit anti-phospho-Smad2 (Ser465/467), 18338 (Cell Signaling Technology, Bever, USA); rabbit anti-NOX2, NB2-41291 and -NOX4, NB110-58849 (Novus Biologicals, USA); rabbit ant-CSE (MyBioSource, USA MBS 769567); rabbit anti-PAX7, ab92317; and the housekeeping genes rabbit GAPDH, ab181602, and histone H3, ab201456 (Abcam, USA).

### 2.10. Western Blot Analysis

Frozen gastrocnemius muscles were powdered in a prechilled mortar under liquid nitrogen, and the resulting materials were sonicated on ice in RIPA buffer containing 1% NP-40, 0.5% deoxycholate, and a protease/phosphatase inhibitor cocktail (Roche Diagnostics), and the resulting homogenates were centrifuged at 15,000 x g for 15 min at 4°C [23]. Supernatant proteins were loaded onto an SDS-polyacrylamide gel and transferred to a PVDF membrane (Bio-Rad). The membranes were blocked and then incubated with the primary antibody diluted in 5% nonfat dry milk in TBST buffer (10 mmol/l Tris-HCl, pH 7.5, 150 mmol/l NaCl, 0.05% Tween 20) overnight at 4°C. After washing, the blots were incubated with the secondary antibody conjugated to HRP in TBST for 1 hour at room temperature. The proteins were visualized with a SuperSignal West Pico Chemiluminescent Substrate (Pierce) according to the manufacturer's protocol.

### 2.11. Statistical Analysis

All results were expressed as mean ± SEM of at least 5 animals/group. The data were compared using one-way analysis of variance and Dunnett's multiple comparison test, or Student *t* test where appropriate. An associated probability of less than 5% was considered significant.

## 3. Results

### 3.1. GK Diabetic Rats Displayed Abnormalities Related to Metabolic Control and Muscle Mass and Strength

**Figure 1:**
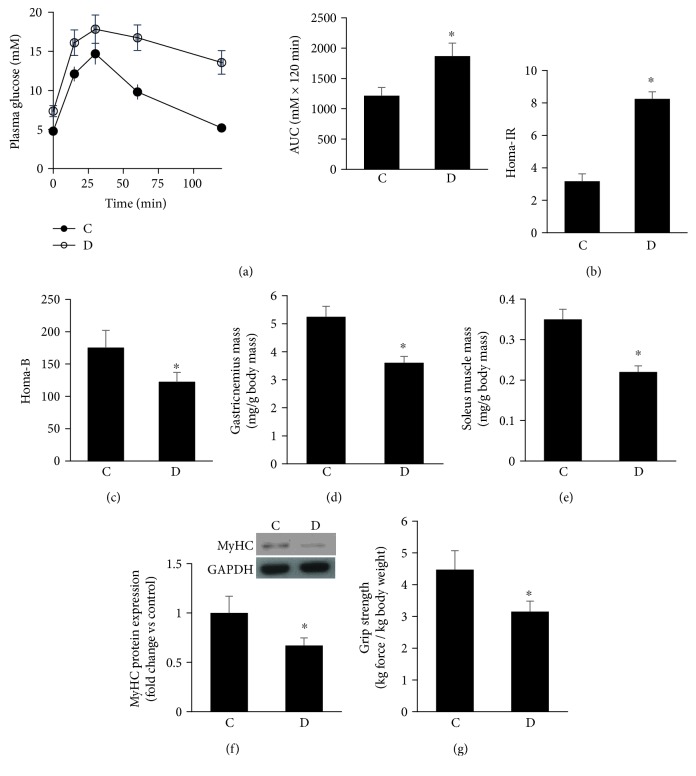
GK diabetic rats displayed abnormalities related to metabolic control and muscle mass and strength. (a) GK rats fasted for 16 h had significantly impaired glucose tolerance as typified by higher AUC values relative to corresponding controls. (b and c) Fasted blood glucose and plasma insulin levels were used to quantify HOMA-IR (b) and HOMA-*β* (c) in control and GK rats. (d and e) Muscles were normalized to body weight. (f) Immunoblot and quantification of MyHC protein in gastrocnemius muscles of control and GK diabetic rats. (g) Grip strength of control and GK rats was normalized to body weight. Abbreviation: C: control; D: diabetic. Values are means ± SEM for at least 6 animals/group. ^∗^Significantly different from corresponding control values at *P* ≤ 0.05.

A 15-month-old GK rat (D) exhibited a characteristic feature typical of type 2 diabetes, and it includes moderate hyperglycemia, impaired glucose tolerance (Figure 1(a)), and insulin resistance, HOMA-IR, assessed based on fasting plasma glucose and insulin levels (Figure 1(b)). In addition, HOMA-*β* used as an index for *β*-cell function showed GK animals to have a significant reduction in *β*-cell function relative to control values (Figure 1(c)).

Next, we measured in GK rats key indices of sarcopenic symptoms with the resulting data revealing a significant reduction in the mass of both gastrocnemius and soleus muscles, even when the data were expressed as a function of body weight (Figures 1(d) and 1(e)).

To determine whether the changes in muscle mass correlates with muscle functionality, a grip strength test was applied on various groups of animals. As shown in Figure 1(f), the diabetic state impacted negatively on muscle functionality. These morphological and biophysical abnormalities harmonized with a data at the molecular level revealing a significant decrease in total muscle protein contents (myofibrillar proteins constitute 50–70%) that is typified by the abundance of MyHC (Figure 1(g)). The contractile protein myosin heavy chain (MyHC) represents the mechanical component of myofilaments. Its alterations are a good indicator for the dynamic balance between protein synthesis and degradation in addition to the functional status of skeletal muscle cells.

It is noteworthy that the aforementioned muscle properties were not altered in the middle-aged control rats (15-month-old) when compared to their corresponding 4-month-young control values (data not shown). Together, these findings support the notion that diabetes accelerates the process of aging and its associated complication of sarcopenia.

### 3.2. Diabetes Alters Key Signaling Molecules Involved in Skeletal Muscle Mass Regulation

**Figure 2:**
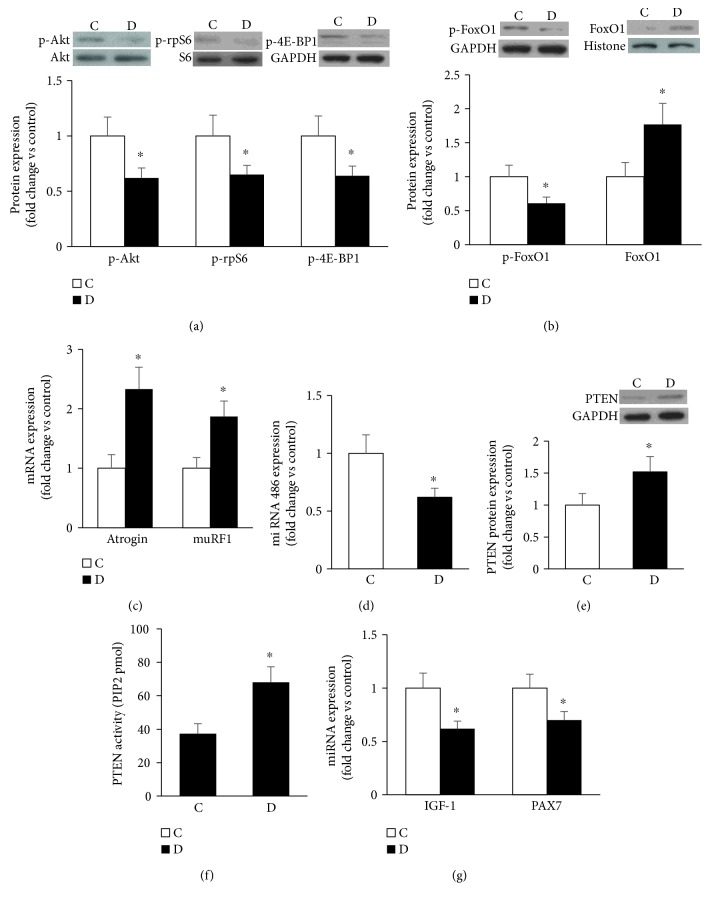
Diabetes alters key signaling molecules involved in skeletal muscle mass regulation. (a) Representative immunoblots and quantifications of phosphorylated Akt, S6, and 4E-BP1 protein levels in muscles of control and GK diabetic rats. (b) Total phosphorylated levels of FoxO1 and its nuclear localization relative to GAPDH and histone, respectively, were analyzed by Western blot. (c) Expression of MuRF1 and Atrogin 1 mRNAs relative to GAPDH was examined by real-time PCR. (d) Expression of miRNA 486 relative to U6 was determined using real-time PCR (e) PTEN protein level (e) and activity (f) were measured as described in Materials and Methods. (f) Key regulators of myogenesis including IGF-1 and PAX7 (g) were assessed in muscle using real-time RT-PCR-based technique. Abbreviation: C: control; D: diabetic. Values are means ± SEM for at least 6 animals/group. ^∗^Significantly different from corresponding control values at *P* ≤ 0.05.

Generally speaking, overall muscle mass is regulated by a delicate balance between protein synthesis and protein degradation with additional contributions from satellite cells and regenerative processes [38]. A major process regulating muscle growth is thought to involve the PI3K/Akt/mTOR-dependent signaling pathway [39]. In this study, we measured the relative level of Akt activation as indicated by Akt phosphorylation at Thr308 and Ser473 with the data showing a significant decrease as a function of diabetes (Figure 2(a); data are shown only for the Ser473 phosphorylation site). Consistent with this finding, we also confirmed a significant decrease in the phosphorylation level of Akt-mTOR targeting molecules including rpS6^ser236/325^ and 4E-BP1^thr37/46^ (Figure 2(a)). Phosphorylation of the latter signaling molecules activates the translational process of protein synthesis.

Next, we sought to examine whether this diabetes-mediated decrease in key regulators of protein synthesis was accompanied by an alteration in the processes involving muscle wasting. In this regard, our initial focus was on the ubiquitin proteasome pathway, primarily because the muscle-specific E3 ubiquitin ligase atrogin-1 (also known as muscle atrophy F-box (MAFbx)) and muscle RING finger 1 (MuRF1) are under the control of an Akt-FoxO-dependent mechanism [38]. The data derived from this study established in diabetic muscle an inverse relationship between the level of Akt-based phosphorylation of FoxO-1 at Thr24 and the nuclear content of FoxO1 protein with a diminution in the former and an elevation of the latter (Figure 2(b)). Interestingly, the downstream target molecules, namely, MuRF1 and atrogin 1 in muscles, were upregulated during the course of diabetes (Figure 2(c)).

Previous reports have shown that in cultured myocardial and muscle cells, miR-486 suppresses protein degradation by reducing FoxO1 activity and PTEN expression [40, 41], a lipid phosphatase that dephosphorylates p-AKT [42]. Accordingly, we sought to examine whether the heightened state of protein degradation in diabetic muscle stems, at least in part, from a mechanism involving the miR-486-PTEN-dependent pathway. Our data using RT-PCR-based assay showed that muscle content of miR-486 was markedly decreased as a function of diabetes (Figure 2(d)). In contrast, an upregulation in PTEN protein expression and activity muscle was evident in this disease state (Figures 2(e) and 2(f)). These abnormalities were associated with a defect in the regenerative and reparative processes as evidenced by the low level of expression of IGF-1 and the satellite cell marker paired box protein 7 (PAX7, Figure 2(g)). Overall, the above findings support the premise that diabetes, by affecting the miRNA 486-PTEN-p-Akt axis, triggers the upregulation of muscle contents of FoxO1 and its downstream molecules atrogin and MuRF. This sequence of event, in the presence of impairment in the adaptive mechanism, is likely to eventuate in a high rate of muscle wasting and consequently sarcopenia.

### 3.3. Diabetic Atrophied Muscles Exhibited a State of Heightened Oxidative Stress (HSOS)

**Figure 3:**
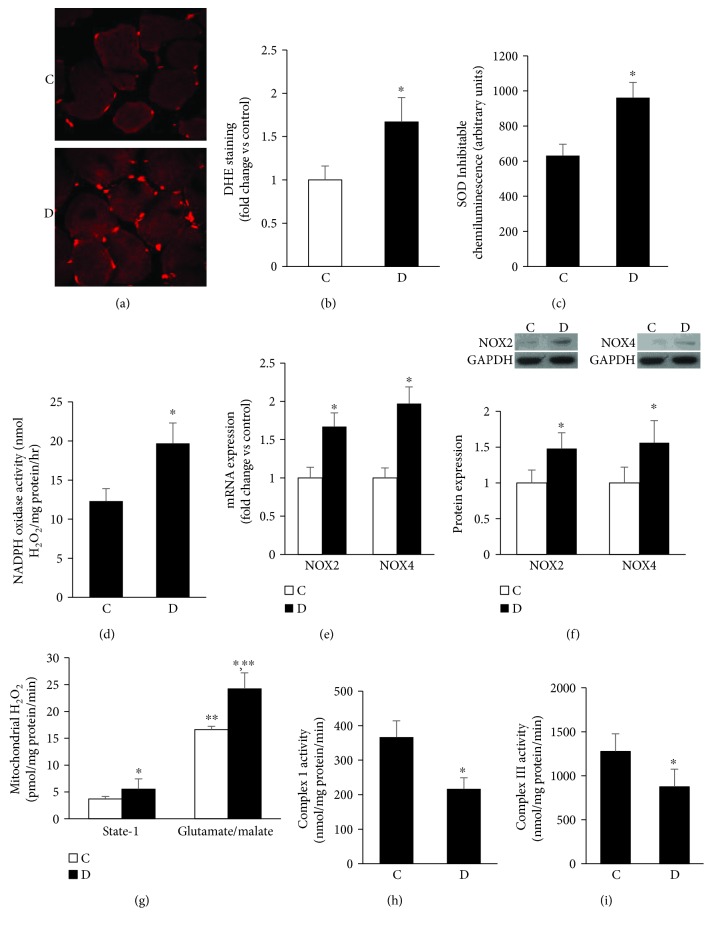
Diabetic atrophied muscles exhibited a state of heightened oxidative stress (HSOS). (a and b) Superoxide generation was measured in frozen muscle sections of control and diabetic using dihydroethidium-based confocal microscopic staining technique. (c and d) NADPH oxidase in a membrane fraction was assessed according to procedure involving the substrate NADPH and lucigenin chemiluminescence or the Amplex Red/horseradish peroxidase fluorescence-based assays. (e and f) Muscle NADPH oxidase-related isoforms including NOX2 and NOX4 were determined at the mRNA (e) and protein levels (f) using RT-PCR and Western blotting-based techniques. (g) Mitochondrial H_2_O_2_ generation at the steady state level and in the presence of added glutamate/malate substrates was measured using the Amplex Red/horseradish peroxidase fluorescence-based assay (g). Activities of complexes I (h) and III (i) of the electron transport chain were measured using spectrophotometric-based assay. Abbreviation: C: control; D: diabetic. Values are means ± SEM for at least 6 animals/group. ^∗^Significantly different from corresponding control values at *P* ≤ 0.05.

Having established that the diabetic state induced muscle atrophy by tipping the balance in favor of muscle-specific protein degradation, we sought to examine whether a common mechanism contributes to the proteasome-mediated activation of the proatrophic-dependent pathway during the course of diabetes. The initial focus was on ROS, which have been shown to be involved in the development of muscle atrophy under different pharmacological and pathological states [43]. Dihydroethidium staining, a relatively specific indicator of superoxide, in a frozen section of diabetic muscle appears to be higher than corresponding control values (Figures 3(a) and 3(b)). This radical is a by-product of enzymatic oxidases and mitochondrial respiration. Accordingly, it was decided initially to test whether the documented elevation in superoxide stemmed from enhanced activity of NADPH oxidase, a multiprotein enzyme complex, which is highly expressed in skeletal muscle and uses NADPH as a substrate to convert molecular oxygen to ROS, usually superoxide or hydrogen peroxide (H_2_O_2_) [44]. In this context, we directly measured the rate of NADPH-dependent superoxide/H_2_O_2_ generation in 100,000 x g membrane fractions of control and diabetic muscles using lucigenin chemiluminescence or the Amplex Red/horseradish peroxidase fluorescence assay. VAS 2870 was used as a specific NADPH oxidase inhibitor [45]. Diabetic muscle produced more superoxide and H_2_O_2_ than corresponding control values, and this finding was associated with a significant increase in the activity of NADPH oxidase (Figures 3(c) and 3(d)). Real time PCR- and Western blotting-based techniques were used to measure the specific isoform of NOX that contributes to diabetes-related increase in muscle ROS formation. Our data revealed that the levels of expression of mRNAs encoding for NOX2 and NOX4, primary subunits of the NADPH oxidase system in skeletal muscle, were augmented in diabetic muscle relative to corresponding control values (Figure 3(e)). Further experimentations were conducted to confirm whether the changes in transcription observed in NOX2 and NOX4 genes are reflected at the level of translation. Immunoblotting was performed with the results showing that the levels of these subunits of NADPH oxidase were augmented in diabetic rats compared with their corresponding control counterparts (Figure 3(f)). To this end, our findings support the premise that the NADPH oxidase system partially contributes to muscle ROS generation during the course of diabetes.

Besides the NADPH oxidase system, the mitochondria also play a key role in ROS production within the cell [46]. Accordingly, we measured ROS production by isolated mitochondria using the Amplex Red-based assay in combination with horseradish peroxidase. This assay is sensitive enough to detect mitochondrial ROS generation even during state I reflecting the absence of exogenously added substrates [47]. The data in Figure 3(g) show an elevation in the level of mitochondrial H_2_O_2_ in diabetic muscle relative to control. Glutamate/malate is a substrate for complex I of the mitochondrial electron transport chain, after which the electrons are transferred to ubiquinone, complex III, complex IV, and ultimately oxygen [48]. Mitochondrial ROS generation in the presence of glutamate in control animals was about 4.3 folds higher than state I; this value was further increased by about 47% in diabetic muscles (Figure 3(g)). Finally, we measured the activities of muscle mitochondrial complexes I and III since these are the major sites where electrons are leaked to oxygen, producing superoxide radicals which go on to form other ROS [46]. We found a significant decrease in complex I (Figure 3(h)) activity and to a lesser extent complex III as a function of diabetes (Figure 3(i)). Together, the above data are consistent with the concept that both NADPH oxidase and mitochondrial dysfunction contribute to the increase in muscle ROS generation during the course of diabetes.

How might the heightened state of oxidative stress mediate the loss of muscle mass? One possibility confirmed above is related to the activation of FoxO-based transcription of atrogin, atrogin 1, and MuRF1, and the second one outlined below involves an impairment in the adaptive mechanism of antioxidant enzymes in connection with the augmented expression of myostatin, a regulator of the balance between catabolic and anabolic processes.

### 3.4. Impaired Antioxidant Capacity and Enhanced Myostatin Expression in Atrophied Diabetic Muscles

**Figure 4:**
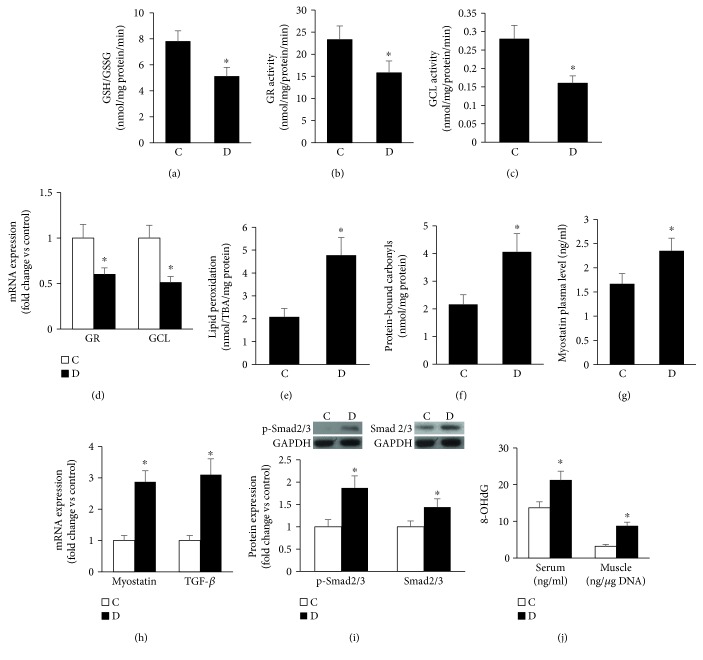
Impaired antioxidant capacity and enhanced macromolecule oxidation in atrophied diabetic muscles. (a–d) Glutathione levels in terms of reduced and oxidized form (a) as well as the activities (b and c) and mRNA expression (d) of key enzymes involved in glutathione metabolism including glutathione reductase and glutamate cysteine ligase were determined using either spectrophotometric or RT-PCR-based technique. (e and f) Oxidation of macromolecules including muscle contents of malondialdehyde (MDA) (e) and protein-bound carbonyls (f), indicators for lipid peroxidation and global protein oxidation, respectively, was measured using commercially available Elisa assays. Myostatin levels in plasma (g) and muscle (h) were assessed as described in Materials and Methods. RT-PCR and Western blot were used to assess TGF-*β* mRNA expression (h) and its downstream signaling molecules Smad2/3 and p-Smad2/3 (i). Serum and muscle levels of 8-OHdG were analyzed by Elisa assays. Abbreviation: C: control; D: diabetic. Values are means ± SEM for at least 6 animals/group. ^∗^Significantly different from corresponding control values at *P* ≤ 0.05.

Heightened state of oxidative stress is dictated not only by the ROS producers but also by the antioxidant defense mechanism exemplified in the current study by the glutathione redox buffering system. This system is a major intracellular buffer of oxidants and is critically involved in protecting cells from ROS-mediated damage. Our data revealed that, in contrast to NADPH oxidase upregulation, the total glutathione and the ratio of GSH (reduced glutathione)/GSSG (oxidized glutathione) were diminished as a function of diabetes (Figure 4(a)). In addition, we also noticed that the activities and expression of key enzymes involved in glutathione metabolism including CSE and glutathione reductase (GR) were also suppressed in muscle of GK rats relative to corresponding control values (Figures 4(b)–4(d)). Contrastingly, muscle contents of malondialdehyde (MDA) and protein-bound carbonyls, indicators for lipid peroxidation and global protein oxidation, respectively, were elevated as a function of diabetes (Figures 4(e) and 4(f)).

We next thought to examine whether the heightened state of oxidative stress noticed in diabetic muscle associates with an elevation in myostatin level, a negative regulator of muscle growth [49] and an activator of the Smad2/3-atrogin-dependent pathway [50]. Our data revealed that myostatin plasma level was elevated as a function of diabetes, and this was corroborated by an elevation in the expression of its mRNA in muscles (Figures 4(g) and 4(h)). Moreover, the expression of TGF-*β* and the levels of its downstream mediator p-Smad 2/3 were likewise increased in this disease state (Figures 4(i) and 4(j)). In contrast, the muscle myostatin receptor, activin 2b receptor, was not altered in GK rats when compared to corresponding control values (0.87 ± 0.14, 0.79 ± 0.16). Interestingly, increased serum content of myostatin in diabetics appears to correlate with a significant elevation in 8-OHdG concentration, an indicator of oxidative DNA damage (Figure 4(k)). To this end, it is possible that diabetes, by increasing ROS generation and reducing antioxidant scavenging capacity, triggers a state of heightened oxidative stress; this phenomenon in turn activates both FoxO-atrogin and myostatin-TGF-*β* related pathways leading to a high rate of muscle wasting and henceforth sarcopenia.

### 3.5. Altered H_2_S Dynamics as a Function of Diabetes

**Figure 5:**
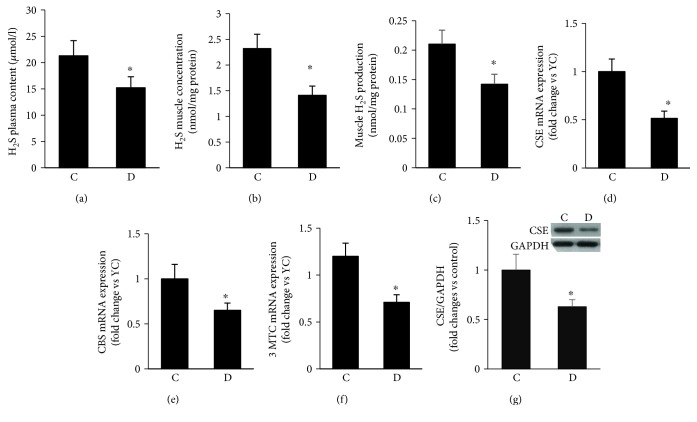
Altered H_2_S dynamics as a function of diabetes. H_2_S levels in serum (a) and muscles (b) together with the rate of H_2_S production (c) were measured using the methylene blue spectrophotometric assay. mRNA expression of key enzymes involved in H_2_S synthesis including CSE, CBS, and 3-MST (d, e, f) was determined using the RT-PCR assay. Western blot was used to measure the protein levels of CSE (f). Abbreviation: C: control; D: diabetic. Values are means ± SEM for at least 6 animals/group. ^∗^Significantly different from corresponding control values at *P* ≤ 0.05.

H_2_S has a powerful antioxidant, antiapoptotic, and anti-inflammatory activities; more importantly, it has been shown recently to be synthesized in skeletal muscles [14]. This gasotransmitter also exerts major effects on mitochondrial function and ATP production [51]. Since some of these H_2_S-based surviving signals are altered and may contribute to sarcopenia during the course of diabetes, it was decided to highlight the H_2_S system in the context of skeletal muscle and diabetes. Plasma and skeletal muscle specimens were collected from various experimental groups, and H_2_S level and production were assessed using a spectrophotometric-based technique. The data revealed a significant decrease in H_2_S concentrations in both plasma (Figure 5(a)) and skeletal muscle (Figure 5(b)) of GK rats, relative to control values. Similarly, generation of H_2_S in skeletal muscle was also reduced as a function of diabetes (Figure 5(c)).

To investigate the mechanisms by which H_2_S generation was downregulated in diabetic muscle, we measured the levels of CBS, CSE, and 3-MST, key enzymes involved in the biosynthesis of H_2_S, at the mRNA level, after confirming that these enzymes are expressed in rat skeletal muscles. Using quantitative real-time PCR, we demonstrated that the expression of mRNA encoding for CSE, CBS, and 3-MST in diabetic muscles were diminished when compared to their corresponding control values (Figure 5(d)). Since CSE displayed the most dramatic changes in response to diabetes and its regulation at the epigenetic level and in response to miRNAs is under consideration in our laboratory, we sought to view this enzyme in the context of protein content using Western blotting-based technique. As depicted in Figure 5(e), diabetes affected CSE protein level in a manner analogous to that seen with mRNA. To this end, our current findings support the notion that all of the aforementioned enzymes are involved in diabetes-induced suppression of H_2_S production in skeletal muscle.

### 3.6. H_2_S Therapy Increases Muscle Mass and Strength and Improves Insulin Sensitivity and *β*-Cell Function in a GK Rat Model of Type 2 Diabetes

**Figure 6:**
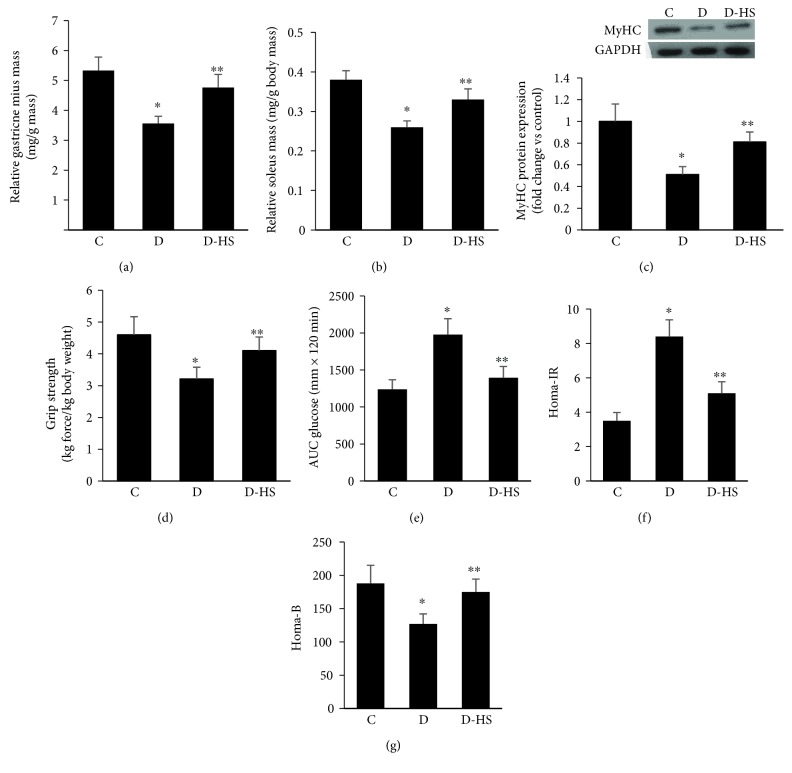
H_2_S therapy increases muscle mass, protein content, and strength and improves insulin sensitivity and *β*-cell function in a GK rat model of type 2 diabetes. Sarcopenic features including muscle mass (a and b), protein level (c), and grip strength (d) in response to NaHS therapy are shown. NaHS improves diabetic metabolic control as indicated by reduced GTT AUC (e), HOMA-IR (f), and an enhancement in *β*-cell function (g). Abbreviation: C: control; D: diabetic. Values are means ± SEM for at least 6 animals/group. ^∗^Significantly different from corresponding control values at *P* ≤ 0.05. ^∗∗^Significantly different from corresponding diabetic vehicle-treated values at *P* ≤ 0.05.

To confirm the involvement of endogenous H_2_S deficiency in diabetes-induced sarcopenic symptoms, we utilized the H_2_S donor NaHS in GK rats. Administration of NaHS daily for one month to diabetic animals restored muscle mass, and protein content (e.g., MyHC) to almost control values (Figures 6(a)–6(c)). Analogously, grip strength, a measure of functionality, was also improved in these animals (Figure 6(d)).

Next, we wanted to know whether the partial amelioration in muscle mass and functionality in NaHS-treated diabetic animals was associated with a similar improvement in diabetic metabolic control. Accordingly, key parameters related to the pathogenetic mechanisms of type 2 diabetes were assessed. The resulting data clearly indicated that NaHS treatment reduced AUC in the GT test (Figure 6(e)), enhanced insulin sensitivity (Figure 6(f)), and induced positive impact on *β*-cell function (Figure 6(g)).

### 3.7. H_2_S Therapy Counteracted Increased Oxidative Stress and Myostatin Levels and Diminished Akt Activity in Diabetic SKM

**Figure 7:**
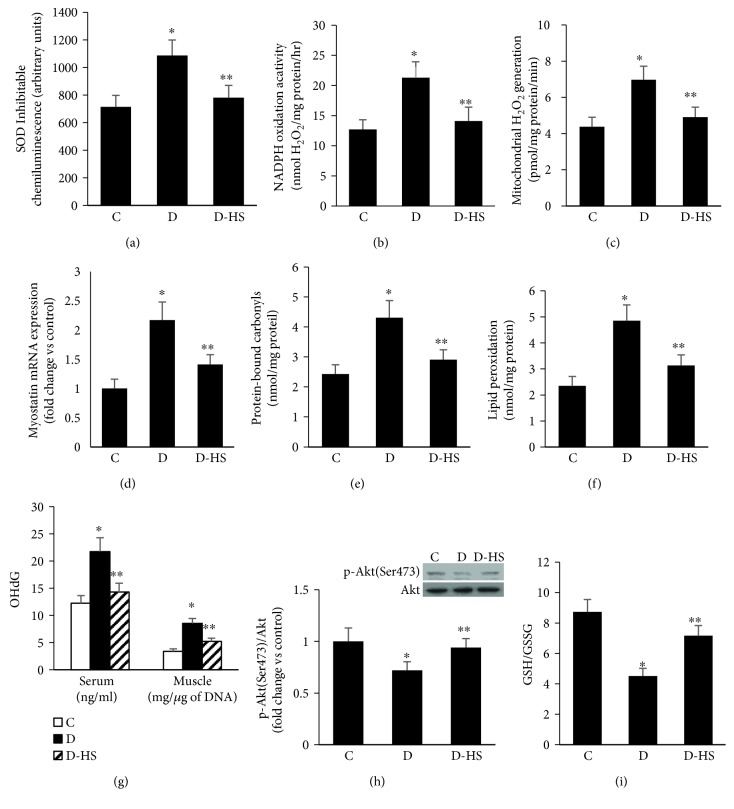
H_2_S therapy ameliorated decreased Akt H_2_S therapy activity and increased levels of myostatin and oxidative stress in diabetic skeletal muscle. H_2_S therapy was given IP to a group of diabetic rats at a dose of 5.6 mg/kg/day for a duration of one month. Muscles were removed and analyzed for Akt activity, myostatin, and oxidative stress. (a–c) Generation of superoxide and H_2_O_2_ in muscle membrane (a and b) (a) and mitochondrial (c) fractions were determined as described in Materials and Methods. (d–g) Myostatin (d) and macromolecule oxidative products including protein-bound carbonyls (e), MDA (f), and OHdG (g) in muscle receiving NaHS therapy were assessed using RT-PCR and ELISA-based assays. (h and i) Akt activity (h) and the antioxidant capacity (i) showed favorable response to H_2_S therapy, and their quantifications were based on spectrophotometric and Western blot techniques, respectively. Abbreviation: C: control; D: diabetic. Values are means ± SEM for at least 6 animals/group. ^∗^Significantly different from corresponding control values at *P* ≤ 0.05. ^∗∗^Significantly different from corresponding diabetic vehicle-treated values at *P* ≤ 0.05.

In view of the current data documenting that NaHS treatment improves the sarcopenic symptoms and metabolic control in diabetics, we sought to further explore whether such beneficial effect of NaHS was associated with a mitigation of the high levels of myostatin and its companion state of oxidative stress. In addition, the master regulator of muscle anabolic and catabolic signaling pathways exemplified by the enzyme activity of Akt was also considered in our study. The data showed that the excessiveness in ROS generation by NADPH oxidase and the mitochondrial organelle (Figures 7(a)–7(c)) together with the parallel increase in levels of myostatin (Figure 7(d)) and macromolecule oxidative products (e.g., protein-bound carbonyls, MDA, and OHdG) were all reduced in muscle of diabetics receiving NaHS therapy (Figures 7(e)–7(g)). In contrast, an augmentation of diabetes-induced decrease in Akt activity (Figure 7(h)) and antioxidant capacity (GSH/GSSG) were also evident in this disease state (Figure 7(i)).

Overall, our study gives credence for the notion that heightened state of oxidative stress in connection with disturbed H_2_S bioavailability may represent novel indicators for the progression of sarcopenia and CLI. Sarcopenia/CLI manifests in multiple pathologies, but therapeutic aim at remedying impaired H_2_S bioavailability with an H_2_S donor may benefit them all.

## 4. Discussion

Sarcopenia is a multifactorial syndrome featured by progressive and generalized loss of skeletal muscle mass and strength with a consequence of impaired functionality. This phenomenon occurs with diabetes and advanced aging, and it may play a role in the development and progression of CLI. The accumulation of ROS and evidence of oxidized lipids and proteins has been implicated in sarcopenic muscle [4]; however, the amelioration of this pathology is contradictory and remains to be explored.

Our data regarding the clinical symptoms of sarcopenia are consistent with those reported previously in mice at 26 months of age and in humans at 60–70 years of age [52, 53]. In a similar vein, type 2 diabetes is considered to be a risk factor for functional disability and for mobility limitation [3]. Indeed, a recent report showed that patients with type 2 diabetes had a threefold higher risk of sarcopenia/frailty than subjects without diabetes [9]. Interestingly and in contrast to nondiabetic aged individual, the deficit in muscle quality (muscle strength/muscle mass) in type 2 diabetic patients starts as young as in their 40s [8]. These findings are not dissimilar from those we obtained in our rat model of type 2 diabetes.

At the cellular and molecular levels, the current study provides evidence of a state of heightened oxidative stress in both mixed (GN) and slow (soleus) muscles of our animal model of type 2 diabetes. Similarly, an upregulation in myostatin expression and the ubiquitin/proteasome system as typified by the FoxO1/MuRF1/atrogin-1 signaling pathway was also confirmed in this disease state. In contrast, a suppression of the PI3K/Akt/mTOR pathways and an impairment in satellite cell activation and proliferation appear to occur during the course of diabetes. These biochemical aberrations were associated with a muscle loss, a deficit in muscle functionality, and a deficiency in systemic and muscle H_2_S bioavailability. Together, the current data point to the possibility that the key features of sarcopenia in type 2 diabetes may stem at least in part from an imbalance between muscle anabolic, catabolic, and regenerative processes. It is noteworthy, illuminating one of the current findings showing that the aforementioned biomechanical and metabolic abnormalities in SKM of type 2 diabetes were ameliorated using a therapeutic strategy involving the antioxidant H_2_S agonist, NaHS.

Oxidative stress representing the net balance of oxidant production to anti-ROS defense has been shown to contribute to the pathogenesis and progression of chronic diabetic complication [22, 23, 26, 54]. The current data showed higher levels of oxidative stress both systemically and in muscles of GK diabetic rats. This phenomenon was evidenced by the excessiveness in mitochondrial and NADPH oxidase-based ROS generation, increased expression of Nox2 and Nox4, and an elevation in plasma levels of DNA oxidative stress marker 8-OHdG. The isoform Nox4 is an O_2_ sensor with a capability of decreasing intracellular concentration of calcium by promoting sarcoplasmic reticulum calcium leak and reducing its uptake [55]. This inability to buffer intracellular calcium may lead to a reduction in the efficiency of skeletal muscle relaxation [56] and alteration in skeletal muscle contraction, thus eventuating in a state of muscle weakness. In view of this information, it is not unreasonable to suggest that diabetes-induced elevation in skeletal muscle Nox4 expression and, henceforth, excessive ROS formation could contribute to the muscle weakness/atrophy observed in this disease state. This proposition is not without precedence since similar data were reported as a function of aging, cancer, and CKD [57–59].

Heightened state of oxidative state occurs in muscles of GK diabetic rats. This phenomenon can be counterbalanced by a net increase in antioxidants including GSH, a tripeptide involved in the maintenance of intracellular redox balance, detoxification, and modulation of critical cellular processes. The present data showed impaired GSH-related antioxidant and detoxification properties during the course of diabetes. These findings are unexpected since under physiological conditions increased ROS are usually mitigated by an adaptive response involving the upregulation of antioxidant enzymes. During the course of diabetes, however, the cellular sensor responsiveness to oxidative stress appears to be impaired and this phenomenon may elicit a high rate of cellular macromolecule oxidative damage. Indeed, our data showing increased muscle contents of malondialdehyde (MDA), and protein-bound carbonyls, indicators for lipid peroxidation and global protein oxidation, respectively, give credence to this suggestion (Figures 4(e) and 4(f)). A case in point in this regard is our unpublished observation demonstrating a defect in the Nrf2 dynamics in diabetic muscle cells. Nrf2 is a key transcription factor that regulates the antioxidant response by inducing the expression of enzymes such as GR and GCL, which contains in their regulatory regions an antioxidant response element (ARE). To this end, the aforementioned phenomenon regarding the defect in the antioxidant adaptive mechanism in diabetes may culminate in a decrease in muscle functionality and an increase in cellular vulnerability toward oxidative stress, as reflected by the elevation in ROS-mediated oxidation of proteins (protein-bound carbonyls), lipids (MDA), and DNA (8-OHdG). Our findings are not dissimilar to those reported previously during aging [60, 61] and CKD [58].

An elevation in ROS and a deficit in the antioxidant response in diabetic muscle may contribute to muscle atrophy through an additional mechanism involving the myostatin/Smad/atrogin-dependent pathway. This premise harmonizes very well with previous reports demonstrating that myostatin, a TGF-*β* family member, negatively regulates muscle growth postnatal development and muscle mass (adulthood) by altering ROS homeostasis, reducing protein synthesis, increasing protein degradation, or impairing muscle regeneration [49, 62–64]. Indeed, myostatin inactivation in mice, through the use of either anti-myostatin antibody (ATA 842; [65]) or a genetically engineered myostatin-neutralizing peptide fused to an Fc fragment (PINTA745; [66]), leads to impressive muscle hypertrophy. Similarly, naturally occurring mutations in myostatin result in a hypertrophic, muscle-bound phenotype in several species such as cows, dogs, and even humans [67–69]. In our GK model of type 2 diabetes, we confirmed an increase in plasma and SKM levels of myostatin and 8-OHdG; a correlation appears that exists between plasma levels of myostatin and 8-OHdG, a known marker of oxidative stress (*r* = 0.71). These results are not unique to the diabetic state since analogous data have been reported in several human diseases associated with skeletal muscle wasting including cancer cachexia, AIDS, heart failure [70], chronic obstructive pulmonary disease [71], and CKD [58].

Myostatin signaling is operative during both development and adulthood. Its cellular actions are mediated in an autocrine/paracrine manner through binding to type II activin receptors A and B (ActRIIA and ActRIIB) which recruit and activate type I activin receptors 4 and 5 (Alk4 and Alk5). These signaling events stimulate Smad2 and Smad3 by phosphorylation of the C-terminal domain. Phosphorylated Smad2/3 can form a complex together with Smad4, and it then moves to the nucleus to trigger gene transcription [72]. We have found in the current study that the muscle contents of pSmad2/3 were increased as a function of diabetes, and this was associated with a similar elevation in the expression of TGF-*β* and atrogin, key players in enhancing proteolysis and MyoD (myogenic transcription factor) degradation during the course of muscle atrophy [50]. To this end, our data support the concept that diabetes may induce muscle wasting and sarcopenic symptoms via activating multiple signaling pathways including the myostatin- and/or TGF-*β*-Smad/atrogin-1-dependent signaling pathway.

Akt regulates both protein synthesis and degradation, respectively, via mTOR and the transcription factors of the FoxO family [50]. On the one hand, Akt enhances protein synthesis of SKM by activating Akt/mTOR and downstream signaling molecules as reflected by the phosphorylation of S6 and 4E-BP1 [73]. On the other hand, Akt promotes the phosphorylation of FoxO1 and consequently decreases the expression of two degradation-associated proteins, namely, MuRF1 and MAFbx [74]. Both high levels of ROS and myostatin appear to inhibit Akt phosphorylation [75, 76]. This is of interest especially when viewed in the context of our current data confirming that the diabetic atrophied muscles are associated with a significant decrease in the phosphorylated forms of Akt, FoxO1, S6, and 4E-BP1 and a marked enhancement in the expression of MuRF1 and atrogin.

H_2_S, a gasotransmitter along with nitric oxide (NO) and carbon monoxide (CO), is generated in mammalian cells including muscle cells by three distinct enzymes, CBS, CSE, and 3-MTS, during methionine/transsulfuration pathways (current study; 14). It affects various physiological and pathophysiological states due to its antioxidative, antiapoptotic, anti-inflammatory and proangiogenic activities [77]. Disturbed H_2_S bioavailability has been reported in a number of diseases such as CLI, hypertension [78], stroke [79], hypercortisolemic state [80], inflammatory disorders [81, 82], and diabetes [17]. However, information regarding H_2_S dynamics in skeletal muscle and its relationship to the pathogenesis of sarcopenia under various pathological conditions and during the course of diabetes is quite limited. Nevertheless, the current data showed that skeletal muscle and serum levels of H_2_S in connection with the expression of H_2_S-producing enzymes were diminished as a function of diabetes, a finding that is consistent with those previously reported in serum, heart, and liver of human and experimental diabetes [83]. A case in point in this regard is the recent data showing that CSE-deficient mice fed a low cysteine diet showed acute skeletal muscle atrophy (myopathy) [84]. Similarly, hyperchromocysteinemia-induced skeletal muscle myopathy was markedly enhanced in mouse model of CBS^−/+^ deficiency [85].

To document that endogenous H_2_S is involved in the loss of muscle mass and functionality during the course of diabetes, we administer to our experimental animals NaHS, a well-known H_2_S donor with proving record for its effectiveness in improving metabolic syndrome in dexamethasone- [80] and high salt-induced hypertension [78]. Analogous with the above findings, we have shown that H_2_S-treated diabetic animals exhibited a significant improvement in the metabolic syndrome as typified by increased insulin sensitivity and enhanced *β*-cell function with partial restoration of the glucose tolerance. Likewise, muscle mass and functionality were also ameliorated in GK rats receiving NaHS therapy that was shown to enhance H_2_S bioavailability. This amelioration in key symptoms related to diabetic sarcopenia was reflected at the molecular level by a reduction in the state of heightened oxidative stress and an enhancement in the so-called Akt activity. Consequently, an augmentation or suppression of the key signaling pathway involved in protein synthesis-(Akt/mTOR, pS6) or degradation-myostatin/FoxO1/MuRF1-atrogin may ensue, leading to the above findings of improvement in muscle mass and functionality.

## 5. Conclusion

Overall, our data support the premise that a relationship exists between sarcopenia and H_2_S deficiency at least in the context of T2D. Given the high prevalence of sarcopenia and muscle insulin resistance in T2D, the current findings advance the notion that the H_2_S system provides attractive molecular targets for the treatment of diabetes-associated muscle dysfunction and diseases involving muscle cell dysregulation. In this perspective, H_2_S donors such as NaHS may be a useful therapeutic strategy in T2D with these common and debilitating conditions, provided that the therapeutic window of H_2_S for a given target is determined in order to minimize the possibility of H_2_S-induced organ toxicity. Indeed, recent advances in H_2_S releasing drugs such as SG1002 for cardiovascular disorders and ATB-346 for arthritis are now progressing into clinical trials and they appear to show considerable promise [86].

## Data Availability

The data used to support the findings of this study are included within the article.
